# Calcium Signaling in Liver Injury and Regeneration

**DOI:** 10.3389/fmed.2018.00192

**Published:** 2018-07-04

**Authors:** Nuria Oliva-Vilarnau, Simona Hankeova, Sabine U. Vorrink, Souren Mkrtchian, Emma R. Andersson, Volker M. Lauschke

**Affiliations:** ^1^Section of Pharmacogenetics, Department of Physiology and Pharmacology, Karolinska Institutet, Stockholm, Sweden; ^2^Department of Biosciences and Nutrition, Karolinska Institutet, Huddinge, Sweden; ^3^Faculty of Science, Institute of Experimental Biology, Masaryk University, Brno, Czechia; ^4^Department of Cell and Molecular Biology, Karolinska Institutet, Stockholm, Sweden

**Keywords:** metabolic disease, non-alcoholic fatty liver disease, ischemic-reperfusion injury, hepatic cholestasis, chronic liver disease

## Abstract

The liver fulfills central roles in metabolic control and detoxification and, as such, is continuously exposed to a plethora of insults. Importantly, the liver has a unique ability to regenerate and can completely recoup from most acute, non-iterative insults. However, multiple conditions, including viral hepatitis, non-alcoholic fatty liver disease (NAFLD), long-term alcohol abuse and chronic use of certain medications, can cause persistent injury in which the regenerative capacity eventually becomes dysfunctional, resulting in hepatic scaring and cirrhosis. Calcium is a versatile secondary messenger that regulates multiple hepatic functions, including lipid and carbohydrate metabolism, as well as bile secretion and choleresis. Accordingly, dysregulation of calcium signaling is a hallmark of both acute and chronic liver diseases. In addition, recent research implicates calcium transients as essential components of liver regeneration. In this review, we provide a comprehensive overview of the role of calcium signaling in liver health and disease and discuss the importance of calcium in the orchestration of the ensuing regenerative response. Furthermore, we highlight similarities and differences in spatiotemporal calcium regulation between liver insults of different etiologies. Finally, we discuss intracellular calcium control as an emerging therapeutic target for liver injury and summarize recent clinical findings of calcium modulation for the treatment of ischemic-reperfusion injury, cholestasis and NAFLD.

## Introduction

The liver serves central functions in the metabolism of lipids and carbohydrates, alcohol, a wide range of drugs, as well as toxins, and is therefore exposed to a diverse set of metabolic insults. Furthermore, the liver is among the most frequent targets of physical injury in abdominal trauma (1). Calcium is an important secondary messenger that is intrinsically involved in a plethora of hepatic processes and, accordingly, dysregulation of calcium signaling is observed across mechanistically diverse injury conditions, including non-alcoholic fatty liver disease (NAFLD) and cholestasis.

To cope with hepatic injuries, the liver has developed a unique ability to regenerate, which might have already been recognized more than two millennia ago, as evident from the myth of Prometheus. However, whether the ancient Greeks indeed knew about the liver's regenerative capacity remains controversial (2). An increasing body of evidence indicates that the liver's regenerative response depends on an accurate orchestration of calcium signaling in both time and space.

In this review, we summarize the current knowledge concerning the role of hepatic calcium signaling across a range of clinically relevant acute and chronic injury conditions. In particular, we explore the role of calcium in acute liver damage due to partial hepatectomy, ischemic reperfusion and drug-induced liver injury (DILI), as well as in chronic liver injury due to metabolic perturbations or cholestasis. In addition, we provide an update of emerging therapeutic strategies that target calcium signaling.

## Calcium signaling in acute liver injury

### Liver regeneration upon physical liver injury

Partial resection of the liver represents a common intervention for patients with hepatic neoplasms, most frequently colorectal cancer metastases and primary hepatocellular carcinomas. During this partial hepatectomy (PHx) up to 70% of the liver is removed, which is followed by regeneration and a full recovery of the initial mass of the organ within few weeks. Due to the inaccessibility of the liver, most mechanistic and time-course PHx data are collected in animal models, primarily rodents. While mature liver cells in homeostatic conditions do not proliferate, after PHx the remaining liver cells reenter the cell cycle, thus regenerating the injured organ (3). This notion was built on seminal studies in rats in which the uptake of radiolabeled nucleotides after PHx was interpreted as the cells′ entry into S phase less than 24 h post operation (4, 5). However, S phase entry does not equal cell division in the case of hepatocytes, as they can be multinuclear and polyploid. Indeed, Miyaoka and colleagues showed that a combination of hypertrophy and unconventional cell proliferation in which binuclear hepatocytes divide and give rise to mononucleated daughter cells accounted for the rapid regenerative response after PHx in mice (6).

Self-renewal of hepatocytes is remarkably slow under homeostatic conditions with cell cycling times between 2 and 4 weeks (7) and only 1 in ~20,000 hepatocytes (0.005%) is in the cell cycle (8). However, upon PHx, hepatocytes rapidly increase their proliferative capacity and reconstitute the liver weight prior to injury within a few weeks (9). Underlying this regenerative response are increases in intrahepatic calcium concentrations during the first days of liver regeneration that parallel cell cycle entry (10). Interestingly, when only one-third of the liver is removed hepatocytes did not enter S-phase and rather recovered the original liver mass by hypertrophic mechanisms (11); however no information about calcium transients is available in this setting.

The transition from rather quiescent to proliferative liver cells is tightly controlled by a complex network of endocrine, paracrine and autocrine signals, including hormones, growth factors, cytokines and bile acids (12). Importantly, transgenic expression of variants of the calcium buffering protein parvalbumin that are targeted to either cytosol or nucleus affects hepatic cell proliferation and impairs liver regeneration *in vivo*, indicating that progression through the cell cycle is dependent on calcium signaling (13, 14).

**Figure 1:**
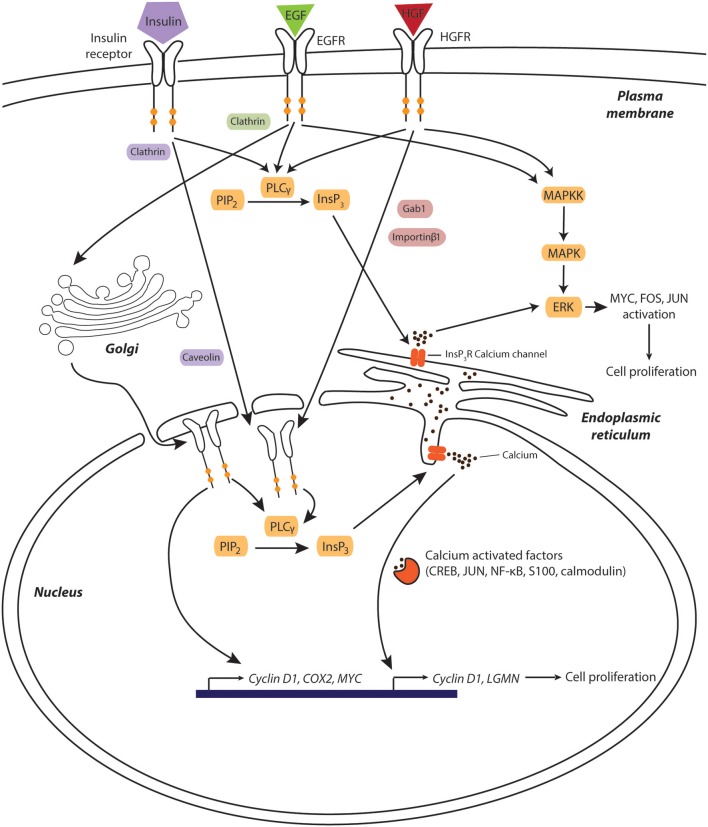
Calcium is a key component of hepatic growth factor signaling during liver regeneration after physical injury. Growth factors induce their mitogenic effects through pathways involving receptor translocation to the nuclear compartment and subsequent triggering of nucleoplasmic calcium release. Increased cytosolic calcium levels activate calcium-dependent transcription factors and kinases, which facilitates cell cycle entry and progression. Furthermore, EGFR directly associates to the promoter of pro-proliferative genes, such as *cyclin D1, COX2*, and *MYC*. Components involved in EGF-, HGF-, and insulin signaling are shown green, red, and purple, respectively. Signaling molecules shared between pathways are depicted in orange.

Epithelial growth factor (EGF) and hepatocyte growth factor (HGF) constitute the most extensively studied mitogens that signal via their receptors, the receptor tyrosine kinases EGFR (also termed ErbB1 or HER1) and HGFR (also termed c-Met), respectively. While endpoints of EGF and HGF signaling do not overlap, inhibition of one or the other pathway delays but does not prevent liver regeneration after PHx (15–18). In contrast, simultaneous ablation of both pathways resulted in liver decompensation (19). Both EGF and HGF elicit signaling via the MAPK-ERK axis leading to phosphorylation and activation of a range of factors that promote cell division, including MYC, FOS and JUN (Figure 1) (20, 21). In addition, calcium signals constitute important mediators of EGF and HGF signaling. The first indications came from experiments in isolated rat hepatocytes in which transient elevations of intracellular calcium levels were detected following EGF or HGF exposure (22). However, the molecular links between growth factor signaling and calcium were only elucidated more than a decade later.

Interestingly, binding of HGF to the HGFR causes the receptor itself to translocate to the nucleus in a process that requires both Gab1 and importin proteins (23). Once inside the nucleus, HGFR activates nuclear phospholipase Cγ (PLCγ), which in turn catalyzes formation of inositol-1,4,5-triphosphate (InsP_3_), opening of InsP_3_-ligand-gated calcium channels and subsequent release of calcium from the nucleoplasmic reticulum into the nucleoplasm (23). The resulting temporary increase in nuclear calcium can directly facilitate the target recruitment of calcium sensitive transcription factors, such as CREB, NF–κB and c-jun, or modulate the transcriptional activity of basic helix-loop-helix transcription factors via the nuclear calcium sensors calmodulin and S100 (Figure 1) (24). One gene whose expression is controlled by nuclear calcium levels encodes the peptidase legumain (LGMN) whose expression was decreased by 97% upon nucleoplasmic calcium buffering, resulting in decreased cyclin expression and impaired proliferation (25).

Similarly to HGFR, upon ligand binding, EGFR can translocate to the nucleus via clathrin and dynamin-dependent endocytosis (26) followed by retrograde translocation via COP1-coated vesicles (27) and shuttling to the inner nuclear membrane via the Sec61β translocon (28). Once in the nucleus, EGFR activates nuclear PLCγ, resulting in nucleoplasmic calcium release (Figure 1). Furthermore, nuclear EGFR can act as a transcriptional coactivator by directly or indirectly binding to promoters of genes essential for liver regeneration, including *CCND1* (encoding Cyclin D1), *COX2*, and *MYC* (29–31).

Insulin is another pro-regenerative factor that stimulates proliferation through nuclear calcium signals. Similarly to HGF and EGF, insulin binds to its receptor tyrosine kinase, which subsequently translocates to the nucleus, activates PLCγ and generates InsP_3_ dependent calcium signals (32). Importantly, this signaling was not only observed *in vitro* but also *in vivo* in hepatectomized rats, where cell proliferation was reduced in animals where nuclear InsP_3_ was efficiently buffered (33). Combined, the presented evidence indicates that liver regeneration depends on nuclear calcium release and that common hepatic mitogens exert their proliferative effect by an interplay of cytoplasmic MAPK activation and signaling along the PLC-InsP_3_-nucleoplasmic calcium axis.

Besides nuclear calcium, mitochondrial, and cytoplasmic calcium signals are putative regulators of liver regeneration. Cytosolic calcium buffering significantly inhibited HGF-induced ERK phosphorylation, resulting in reduced levels of cyclin expression and retinoblastoma protein phosphorylation, as well as delayed DNA synthesis and reconstitution of liver mass after PHx in rats (14). In contrast, mitochondrial calcium buffering resulted in accelerated liver regeneration, possibly by inhibition of apoptosis (34). Corroborating this study, hepatic loss of *MICU1*, a calcium-sensing regulator of the mitochondrial calcium uniporter, results in mitochondrial calcium overload upon physiological stress conditions and susceptibility to mitochondrial permeability transition pore (MPTP) opening, which leads to the abrogation of hepatocyte proliferation and extensive necrosis after PHx in mice (35).

In addition to the recognized roles of intracellular calcium signaling, extracellular calcium transients can affect hepatic signaling and liver regeneration. The Notch pathway can act as a sensor of extracellular calcium concentrations in cancer cells and during development (36, 37). Mechanistically, calcium depletion dissociates the Notch receptor heterodimer (38), releasing the Notch intracellular domain (NICD), which subsequently translocates to the nucleus and activates transcription of target genes together with the transcriptional coactivator RBPJκ (39, 40). Importantly, Notch-signaling has been recently implicated in the stimulation of hepatocyte proliferation during liver regeneration after PHx (41). Thus, while a direct role of extracellular calcium levels in liver regeneration has not yet been demonstrated, it is conceivable that extracellular calcium levels partake in the orchestration of the regenerative response and this regulation remains an interesting area of future research.

Notably, while human hepatocytes readily proliferate within the liver stroma, this capacity is lost upon culture *ex vivo*. In conventional 2D monolayer cultures primary human hepatocytes (PHH) rapidly lose their hepatic phenotype and functionality with the first transcriptional changes being detectable as early as 30 min after the start of culture (42). Furthermore, PHH in 2D culture form actin stress fibers, resulting in fibroblast-like cell morphology (43). Due to these limitations, only few studies have reported long-term culture and proliferation of hepatocytes *in vitro*. Rodent cells were described to proliferate up to 35-fold when co-cultured with a fibroblast feeder-layer over 2 weeks (44). Moreover, in a recent study, Katsuda and colleagues showed that primary rat hepatocytes stimulated to proliferate using a small molecule cocktail of ROCK and TGFβ inhibitors and Wnt-/β-catening-signaling activators and EGF could undergo at least 26 passages in long-term culture (45). However, whether calcium signaling was involved in proliferation also in long-term culture was not evaluated. It would thus be of interest to elucidate whether hepatocytes can remain responsive to calcium cues in the long-term and, consequently, design optimal treatment regimens to sustain proliferation.

In recent years, much progress has been made in developing hepatic *in vitro* culture paradigms in which PHH maintain their functionality in culture for extended time periods, thus facilitating faithful modeling of *in vivo* liver function. These include sandwich cultures, culturing PHH in spheroid conformation and various chip- and bioreactor-based culture systems (46). While some of these platforms successfully extended the functional life span of liver cells for multiple weeks, none of these platforms have been successfully applied to expand PHH in culture and further research is thus needed to comprehensively describe the role of calcium in these culture systems.

Taken together, these data demonstrate that a tightly balanced interplay of nuclear, cytoplasmic and mitochondrial calcium signals controls liver regeneration after physical injury. Nuclear calcium transients caused by translocation of activated receptor tyrosine kinases appear to be essential drivers of the liver's regenerative response. Moreover, cytosolic calcium appears necessary to support activation of the HGFR-MAPK-ERK cascade. Thus, the compartmentalization and spatiotemporal regulation of calcium signaling plays an essential coordinating role in liver regeneration.

### Interplay between liver regeneration and hepatic metabolism

Calcium levels contribute to the control of hepatic metabolism, which in turn constitutes an essential factor for liver regeneration (47). In PHx, the remaining liver lobules rapidly increase gluconeogenesis within 30 min to counteract the acute hypoglycemia caused by the loss of large amounts of glycogen and gluconeogenetic capacity (48). Interestingly, hypoglycemia appears to be a necessary cue for the initiation of the regenerative response, as glucose supplementation impairs liver regeneration, whereas caloric restriction prior to PHx facilitates liver cell proliferation (49, 50). Underlying these observations are findings that both genetic and diet-induced hyperglycemia reduce expression and activity of EGFR (51, 52).

Induction of gluconeogenesis is coordinated with a slightly delayed (12–24 h after PHx) redistribution of lipids from adipose tissue toward the liver, resulting in transient hepatic lipid accumulation (53, 54). As with hypoglycemia, suppression of steatosis impairs the regenerative response (53, 55, 56). In contrast, preexisting steatosis strongly associates with postoperative complications and mortality after hepatic resection (57).

In sum, these studies suggest that modulations of hepatic metabolism constitute an essential early event during liver regeneration. As indicated by the essential role of transient hypoglycemia, metabolic alterations do not only seem to fulfill the energetic needs of the regenerating liver, but rather seem to be an independent cue that stimulates growth factor signaling.

### Calcium signaling in hepatic ischemia-reperfusion injury

Hepatic ischemia-reperfusion injury (IRI) can arise during liver surgery, due to hepatic trauma or disruption of the sinusoidal microcirculation and constitutes a major determinant of graft function (58). Mechanistically, IRI consists of two phases, an early ischemic insult, characterized by metabolic perturbations, hypoxia and ATP depletion, and an inflammatory reperfusion injury mediated mostly by Kupffer cells that become activated upon response to hepatocellular damage signals (59, 60). Hypoxia impairs mitochondrial respiration and ATP synthesis, results in an increased production of reactive oxygen species (ROS) and calcium release from the ER into the cytosol (61). The resulting elevated cytosolic calcium levels cause increased calcium uptake into mitochondria and mitochondrial calcium overload, which in turn leads to MPTP opening, mitochondrial depolarization and the initiation of cell death (62, 63). Importantly, genetic ablation of the MPTP component cyclophilin-D protects mice from cell death due to calcium overload, oxidative stress, and IRI (64, 65). Thus, inhibition of mitochondrial calcium uptake or MPTP opening present compelling approaches to ameliorate IRI.

Inhibition of mitochondrial calcium uptake using ruthenium red in a rat model of IRI significantly decreased liver injury markers by 3-fold after injury (66). While these findings clearly demonstrate the central role of calcium in hypoxic liver injury, ruthenium red itself does not provide a viable therapeutic strategy to prevent IRI due to its lack of specificity. Preventing calcium release from the endoplasmic reticulum (ER) using the ryanodine receptor antagonist dantrolene resulted in improved morphological maintenance of hepatic endothelial cells, the main target of IRI (67).

In addition to direct calcium modulations, preoperative treatment of patients with antioxidants, which reduce ROS and calcium release from the ER, can reduce the levels of liver injury markers and decrease the length of stay in intensive care (68). While positive effects were observed with α-tocopherol, no clinical benefits were observed with N-acetylcysteine (69). Another strategy is the direct prevention of calcium-induced MPTP formation or opening by edavarone, which successfully protects from IRI in rat and dog liver resection models (70, 71). Moreover, inhibition of MPTP by cyclosporine A reduces IRI in patients undergoing percutaneous coronary intervention (72); however, clear results in liver transplantation are lacking. For a more comprehensive overview of the topic we refer the interested reader to excellent recent reviews (73, 74). Thus, while multiple promising strategies have been identified in recent years, relatively little progress has been made in the clinical translation of these findings in the context of hepatic IRI and therapeutic guidance from randomized prospective studies is currently lacking.

### Drug-induced liver injury

Drug-induced liver injury (DILI) is an important adverse drug reaction and remains the prime reason for post-marketing withdrawals (75), including the notable examples of troglitazone, ximelagatran, lumiracoxib, and sitaxentan. In the clinics, the vast majority of DILI cases are attributable to acetaminophen (APAP) overdoses (76). In addition, despite much progress in pharmacogenetic biomarker discovery (77), unpredictable, idiosyncratic DILI events have been reported for at least 470 marketed medications approved by the FDA (78), of which chlorpromazine, azathioprine, sulfasalazine, diclofenac and amoxicillin-clavulanic acid are the most common (79). In recent years there is moreover an increasing number of cases of liver injury due to herbal and dietary supplements (HDS), by now accounting for 20% of all reported hepatotoxicity cases in the US (80).

Drugs and HDS can induce liver injury by various mechanisms that differ by the affected hepatic cell type, molecular target, dependency on metabolic activation or the involvement of the immune system. In the frame of this review we focus on liver injury events that involve the disruption of intracellular calcium homeostasis, particularly ER stress and mitochondrial depolarization. For further mechanisms that are outside the scope of this review, we refer the interested reader to recent comprehensive reviews (81–83).

Drug-induced ER stress and the resulting calcium imbalance have emerged as an important event in DILI (84). APAP causes hepatotoxicity via its reactive metabolite *N*-acetyl-*p*-benzoquinone-imine (NAPQI) that depletes cellular glutathione and covalently binds to proteins, resulting in mitochondrial dysfunction, oxidative stress and hepatic necrosis (85). NAPQI also causes ER stress by binding to the ER-resident proteins glutathione-S-transferases, protein disulfide-isomerase (PDI), calreticulin, and SERCA (86, 87). PDI and calreticulin play major roles in protein folding, and covalent binding of NAPQI thus reduces protein folding capacity and triggers ER stress and the unfolded protein response (UPR). Notably, while genetic ablation of the UPR effector CHOP in mice had protective effects on APAP overdose-induced lethality, the delay in onset of ER stress compared to mitochondrial dysfunction and elevations in calcium concentrations indicates that ER stress is a clinically relevant but secondary effect of APAP hepatotoxicity (88).

Diclofenac, a non-steroidal anti-inflammatory drug (NSAID), is a widely prescribed inhibitor of the cyclooxygenases COX1 and COX2 that inhibits the production of prostaglandins and prostanoids. Metabolism of diclofenac generates reactive *p*-benzoquinoneimines, which can bind to ER proteins causing ER stress and subsequent increases in cytosolic calcium levels (89, 90). Interestingly, chelation of intracellular calcium or inhibition of the InsP_3_R drastically decreased diclofenac-induced hepatotoxicity in HepG2 cells *in vitro*, indicating that calcium release from ER stores is a key event in diclofenac-induced liver injury (91). Similarly, exposure to the antiretrovirals efavirenz, ritonavir, and lopinavir caused ER stress and cytosolic calcium elevations (92, 93). Ritonavir and lopinavir caused inhibition of the ER-resident calcium uptake transporter SERCA, redistribution of endoplasmic calcium into the cytosol and, in the presence of additional hepatic insults such as ethanol, subsequent influx of calcium into the mitochondria, resulting in hepatotoxicity (92). In contrast, efavirenz primarily targets the mitochondria, resulting in mitochondrial depolarization, increased ROS production and shedding of calcium into the cytosol (93). Thus, efavirenz-induced ER stress appears to be a secondary effect of mitochondrial dysfunction.

These findings indicate that drug-induced mitochondrial injury and ER stress mutually affect each other with perturbations of calcium signaling as a shared central hallmark. Further corroborative evidence comes from studies with the experimental SERCA inhibitor thapsigargin, a prototypical inducer of ER stress that causes depletion of ER calcium, increases in cytosolic and mitochondrial calcium levels and subsequent MPTP opening (94). Mitochondria are tightly associated with the ER at specialized subdomains, termed mitochondria-associated membranes (MAM), facilitating the rapid transmission of calcium ions (95). Under ER stress, the protein composition at these synapses changes drastically and promotes mitochondrial calcium overload and apoptosis, mediated in part by the truncated SERCA isoform S1T that causes increased calcium leakage (96).

Mitochondria are a common target of drug toxicity and mitochondrial injury constitutes an important mechanism of DILI (97). Hepatotoxic drugs can cause mitochondrial dysfunction through various mechanisms but most frequently damage occurs via MPTP opening, cytochrome c release and subsequent activation of effector caspases. Notable examples of drugs inducing mitochondrial permeability transition are salicylic acid, nimesulide, disulfiram, valproic acid, troglitazone, and alpidem. In turn, elevated extra-mitochondrial calcium concentrations can aggravate MPTP opening (98), thus linking increased hepatic stress to increased susceptibility to drug-induced hepatotoxicity. However, elevated mitochondrial calcium concentrations can trigger moderate cytochrome c release in hepatocytes, even without MPTP opening (99). In the cytoplasm, the released cytochrome c can bind to InsP3 receptors on the ER which causes release of calcium from ER stores thus further amplifying the apoptotic signal (100).

Combined, these studies point to a central role of calcium and ER-mitochondrial crosstalk in the orchestration of drug-induced apoptosis. Importantly, blocking of MPTP formation or opening by cyclosporine A may be protective against hepatotoxicity induced by these drugs. Furthermore, it will be interesting to see whether therapeutic strategies can be developed to directly target the MAM and inhibit liver cell apoptosis, e.g., by blocking calcium export via InsP3 receptors or specific inhibition of S1T synthesis or action.

## Calcium signaling in chronic liver injury

**Figure 2:**
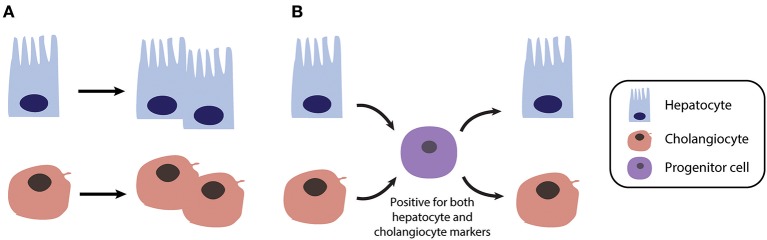
Different mechanisms of liver regeneration in response to acute and chronic liver injury. **(A)** Regeneration after acute injury, as modeled in 2/3 partial hepatectomy, is primarily driven by self-duplication of all parenchymal cell types (mainly hepatocytes and cholangiocytes). **(B)** In contrast in chronically injured liver, such as in NAFLD or cholestasis patients, regeneration can occur via transient bipotent progenitor cells.

In contrast to acute liver damage in which liver regeneration is driven by self-duplication of mature hepatocytes, chronic liver injury entails liver regeneration via distinctly different mechanisms that appear to involve facultative stem or progenitor like cells (Figure 2) (101–103). The first indications of stem cell-mediated regenerative processes were obtained in hepatectomized rats upon administration of 2-acetylaminofluorene (AAF), a chemical compound which prevents hepatocyte proliferation by inhibiting DNA synthesis. In this setting, progenitor cells termed “oval cells” (due to their oval nuclei and sparse cytoplasm) proliferate at the Canals of Hering and may give rise to mature hepatocytes that repopulate the organ (104).

In humans, oval cells are commonly termed hepatic progenitor cells (HPCs) and are observed in “ductular reactions,” an emerging structure observed in a range of chronic liver injuries, including non-alcoholic steatohepatitis (NASH) (105, 106) and cholestasis (107). However, in stark contrast to prototypic stem cell populations in other tissues, such as hematopoietic progenitors or stem cells in intestinal crypts, hepatic progenitor cells have not been found in healthy liver. This has led to a long-standing debate regarding the origin and identity of HPCs. Multiple alleged marker signatures of HPCs have been presented over the years. While the exact markers differ between studies and injury models, a common theme is that HPCs are characterized by a bivalent expression signature, expressing markers of both hepatocytes (e.g., HNF1α, HNF1β, and CEBP) and biliary cells (e.g., SOX9, EpCAM, and CK19) (108).

Particularly SOX9, a transcription factor expressed in cholangiocytes but not hepatocytes, that is associated with the maintenance of a dedifferentiation cell state in multiple contexts (109, 110), appears promising. In a landmark study, Furuyama et al. identified a small Sox9^+^ self-renewing cell population that was capable of supplying hepatocytes in both physiological and diet-induced injury conditions in mice (111). The utility of Sox9 as a marker for progenitor cells was supported by a later study, which suggested that regeneration in a chemically-induced model of chronic liver injury was driven by a subset of periportal Sox9^+^ hepatocytes (112). Intriguingly, Sox9 activity depends on its calcium-dependent binding of calmodulin and inhibition of this interaction abolishes nuclear import and transcriptional activation of Sox9 target genes (113).

In contrast, two recent lineage-tracing reports argued against a role of putative stem or progenitor cells in several diet-induced mouse models of liver injury. One study pulse-labeled hepatocytes in reporter mice and found that contribution of non-labeled cells to the repair of injury was found to be negligible (114). Similarly, no labeled hepatocytes were found after pulse labeling of cholangiocytes again suggesting liver regeneration driven by hepatocyte self-replication (115). However, it remained unclear whether the utilized models indeed are reflective of chronic liver injury.

Thus, these studies could not dismiss the possibility that mature hepatocytes or cholangiocytes may act as facultative stem cells in cases of severe chronic liver injury when cell proliferation is depleted (Figure 2). Indeed, elegant studies using genetically labeled hepatocytes and serial transplantations revealed that both mouse and human hepatocytes can undergo reversible ductal metaplasia in response to injury thus corroborating the concept of liver cell plasticity (116). Similarly, when labeled, replication-deficient hepatocytes were traced in different chronic injury settings, non-labeled cells were found to give rise to new hepatocytes (117). These new hepatocytes were adjacent to cells of biliary origin and were positive for both hepatocyte and biliary markers (117). Furthermore, hepatocytes can serve as a source of functional cholangiocytes and reconstruct peripheral bile ducts in a Notch signaling liver knockout mouse model of Alagille syndrome (118).

Combined, these data lean towards a model of liver regeneration in which both hepatocytes and biliary cells are capable of switching phenotypes via transitioning through dedifferentiated bipotent intermediates when circumstances require so. Importantly while the regenerative response is different, chronic liver injury also directly involves calcium perturbations and inhibition of correction of these perturbations provides emerging therapeutic strategies for a variety of liver diseases.

### Calcium signaling in metabolic disease

**Figure 3:**
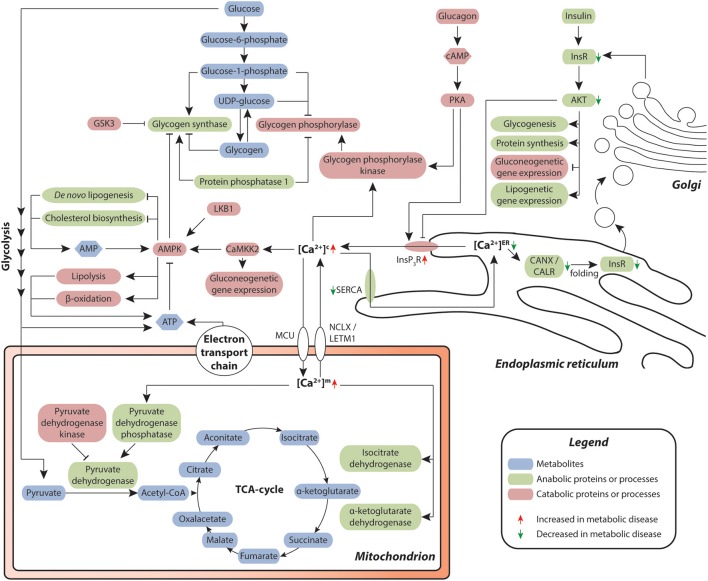
Hepatic calcium signaling in metabolic disease. Cytosolic calcium is of central importance for the orchestration of metabolic control in the liver. Chronic redistribution of calcium from the ER into the cytosol results in a diminished capacity of calcium-dependent folding in the ER, resulting in ER stress and unfolded protein response. Signaling via the Insulin-InsR-AKT axis contributes to a decrease of calcium efflux from the ER. As a result, less correctly folded insulin receptor (InsR) is produced, leading to reduced insulin signaling and reduced AKT-mediated negative feedback regulation of InsP_3_R-dependent calcium efflux from the ER, thus reinforcing insulin resistance. Metabolites are depicted in blue, proteins and secondary messengers involved in anabolic and catabolic processes are shown in green and red, respectively.

Metabolic syndrome is defined as a cluster of interconnected physiological, clinical, and metabolic factors, including hypertension, abdominal adiposity, hyperglycemia, insulin resistance, and dyslipidemia (119). Development of metabolic syndrome is fueled by dietary habits, lack of physical activity, smoking, as well as physiological and genetic factors that perturb metabolic homeostasis and reciprocally promote the development of an array of pathologies, such as type 2 diabetes mellitus (T2DM) and NAFLD (120, 121). In recent decades, multiple lines of evidence demonstrated that calcium signaling is a key regulator of nutrient uptake, metabolism, and utilization (122) and as such provides a critical link between nutritional overload, metabolic dysregulation and hepatic injury (Figure 3) (123).

In the liver, metabolic disease manifests as steatosis and reduced insulin sensitivity. Hepatic hyperlipidosis results in an imbalance between the ER membrane lipids phosphatidylcholine and phosphatidylethanolamine, which impairs the functionality of the endoplasmic calcium uptake pump SERCA (124). Combined with the reduced insulin-dependent control of ER-resident InsP_3_R calcium channel opening in the livers of dyslipidemic, insulin resistant patients, these perturbations lead to elevated cytosolic and reduced endoplasmic calcium levels. (125–127). Importantly, in the ER, calcium is necessary for the functionality of calcium-dependent molecular chaperones, including calnexin (CANX) and calreticulin (CALR), which control the folding of secretory and membrane proteins, including insulin receptors (128). Thus, low calcium levels in the ER entail an overload of misfolded proteins, resulting in ER stress.

In response to ER stress, hepatocytes initiate the UPR in an attempt to reestablish normal ER function by initiating a spectrum of adaptive signaling pathways including increased expression of chaperones. Importantly, overexpression of hepatic calcium-dependent ER chaperones, such as GRP78 (also termed HSP5A or BiP) or ORP150 (also termed HYOU1 or GRP170) can ameliorate hepatic *de novo* lipogenesis and improve insulin sensitivity in genetically and diet-induced mouse models, likely by increasing insulin receptor expression (129, 130). Conversely, hepatic chaperone knock-downs decrease insulin sensitivity in the liver (130).

The UPR can moreover cause insulin resistance directly through different pathways. Firstly, UPR activates the ER stress sensor inositol-requiring enzyme 1 (IRE1; encoded by the *ERN1* gene) (131), resulting in increased JNK activity, which suppresses signaling through the insulin receptor, thereby reinforcing the disruption of calcium homeostasis (132). Secondly, UPR causes upregulation of the tribbles homolog 3 (TRB3), which inhibits signaling via the insulin receptor axis (133, 134). In addition to insulin resistance, elegant studies using a genetic mouse model of impaired ER calcium reuptake by genetic ablation of the SERCA activator *Cisd2* have implicated reduced ER calcium levels and resulting ER stress in the development and progression of NAFLD (135).

Besides its role in the ER, elevated calcium concentrations in the cytosol result in pronounced metabolic reprogramming by activating the calcium-sensitive regulatory δ-calmodulin subunit of glycogen phosphorylase kinase, which in turn stimulates glycogenolysis by phosphorylating glycogen phosphorylase (125). Furthermore, cytosolic calcium activates the Ca^2+^/Calmodulin-dependent protein kinase CaMKK2 (136), which phosphorylates and thereby stimulates AMPK (137), a central regulator of metabolic homeostasis, controlling protein, lipid, and carbohydrate metabolism. AMPK, in turn, inhibits anabolic processes, such as glycogenesis, *de novo* lipogenesis and cholesterol biosynthesis by inhibitory phosphorylation of the central metabolic enzymes glycogen synthases, acetyl-CoA carboxylases and HMG-CoA reductase, respectively (138).

Inversely, catabolic processes, such as glycolysis as well as lipolysis and β-oxidation, are induced. Furthermore, CaMKII activates FOXO1, a central transcription factor controlling expression of gluconeogenetic genes (139), which results in an alignment of the AMPK-dependent stimulation of glycogenolysis with the transcriptional activation of gluconeogenesis (140, 141). Moreover, the increase of cytosolic calcium levels results in the preferential binding of calcium to phosphoinositides, which blocks the insulin-induced recruitment of AKT to the plasma membrane (142). Combined with ER stress-dependent reduction in membrane-bound insulin receptor levels described above, these events lead to a blunting of insulin signaling and, via failing inhibition of InsP_3_R, progressive insulin resistance.

Elevated cytosolic calcium levels furthermore impact on mitochondrial calcium concentrations. Mitochondrial calcium concentrations are controlled by the coordinated interplay of the mitochondrial calcium uniporter (MCU) holocomplex (143–145) and the Na^+^ or H^+^/Ca^2+^ antiporters NCLX and LETM1 (146, 147). Seminal work by Richard Denton and colleagues demonstrated that within the mitochondria, calcium signals directly stimulate pyruvate dehydrogenase phosphatase, which activates pyruvate dehydrogenase and results in an increased rate of acetyl-CoA synthesis (148). In addition, increases in mitochondrial calcium levels result in allosteric modifications of key enzymes of the citric acid cycle (149, 150). As a result, ATP synthesis is increased, which in turn decreases AMPK activity (151) and thus provides a negative feedback loop that assures energy homeostasis. However, in metabolic disease with chronically elevated cytosolic calcium levels, calcium uptake via the MCU pore is increased, whereas export via the calcium transporters becomes saturated (152). This overload of the mitochondrial calcium buffer capability leads to an elevation of mitochondrial calcium levels, increased ROS production and mitochondrial stress, which eventually results in MPTP opening, mitochondrial depolarization, cytochrome c release and apoptosis (63, 153).

Targeting ER stress and UPR constitute emerging therapeutic approaches to improve insulin sensitivity in diabetic patients. AMPK is a central regulator of energy homeostasis and as such an attractive target for the treatment of metabolic disorders. Metformin, the first-line therapy for type 2 diabetes mellitus (T2DM), likely acts via inhibition of complex I in the mitochondrial respiratory chain (154, 155), which results in an increased AMP-to-ATP ratio. This change in energy balance activates AMPK, causing inhibition of anabolic processes and activation of lipolysis and β-oxidation (156), which results in improved lipid profiles and enhanced SERCA activity. Consistent with these effects, metformin relieves ER stress (157, 158) and antagonizes insulin resistance by supporting insulin receptor folding and inhibiting ER stress-induced activation of gluconeogenesis (159). However, metformin can also act in an LKB1- and AMPK-independent manner, as gluconeogenesis was suppressed upon metformin treatment in mice lacking either of the kinases in the liver (160). Besides metformin, an array of direct and indirect AMPK activators are on the market or in clinical development (161).

Besides reducing ER stress via activation of AMPK, innovative strategies are emerging that involve direct targeting of UPR. The chemical chaperones sodium phenylbutyrate and tauroursodeoxycholic acid (TUDCA) reduce ER stress and normalize insulin sensitivity, hyperglycemia, and NAFLD in mouse models (162). Based on these promising results, both drugs were taken forward into the clinics with three trials already completed (NCT00771901, NCT00533559, and NCT03331432) and further trials with TUDCA are currently ongoing. Clinical results align with preclinical mouse data and indicate that both compounds have positive effects on insulin sensitivity in diabetic patients without significant adverse reactions (163, 164). In addition, TUDCA is also used for treatment of cholestasis and primary biliary cirrhosis, as detailed below. In addition, stimulation of hepatic SERCA activity by the small molecule azoramide resulted in an improved protein-folding capacity of the ER and alleviation of ER stress and insulin sensitivity in obese mice (165).

Combined, the presented data demonstrate that calcium signals take up a central role in hepatic energy homeostasis. In recognition of these findings, novel therapeutic strategies are emerging particularly for T2DM that aim at improving hepatic steatosis and insulin sensitivity by directly targeting the intracellular calcium balance.

### Cholestasis

**Figure 4:**
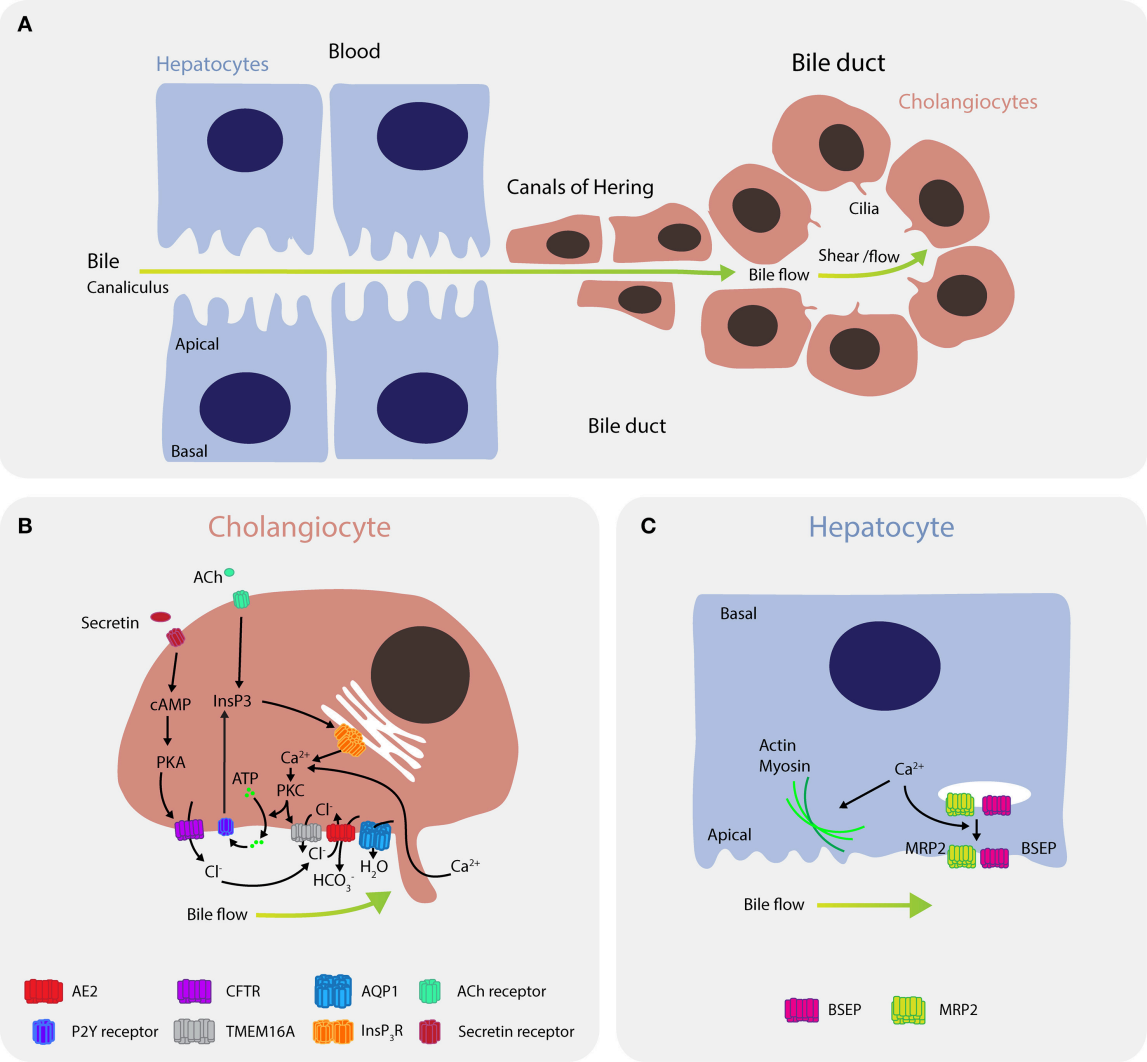
Calcium signaling regulates bile flow in hepatocytes and cholangiocytes. **(A)** Bile is secreted by hepatocytes into the canaliculus where it is transported to canals of Hering and then into bile ducts. **(B)** In cholangiocytes, calcium is predominantly released through the type III isoform InsP3R channels (ITPR3) in response to signaling via M3 muscarinic acetylcholine receptors (CHMR3) and purinergic receptors P2. Furthermore, mechanical cues, such as bile flow and shear stress, induce calcium uptake from the bile through ion channels in the mechanosensory primary cilium. Bicarbonate (${\text{HCO}}_{3}^{-}$) exchange through the anion exchange protein 2 (AE2) depends on extracellular chloride concentrations (Cl^−^), which are regulated by TMEM16A and CFTR. Calcium activates the chloride channel TMEM16A to mediate chloride-bicarbonate exchange at the apical side, while CFTR signaling is activated when secretin binds the secretin receptor on the basolateral membrane, leading to formation of cAMP, activation of PKA and efflux of chloride through the phosphorylated CFTR. Downregulation of ITPR3 in cholestasis, which severely disrupts calcium signaling in cholangiocytes, is thought to be a key mechanism determining bile flow and pathology. Dysregulation of calcium signaling affects multiple pathways regulating both secretion and bile flow. **(C)** In hepatocytes, calcium is affected by ATP, angiotensin II, vasopressin, glucagon, or epinephrine, and regulates actin-myosin contractility to control peristaltic contractions, as well as exocytic insertion of the bile salt export pump (BSEP) and the multidrug resistance-associated protein 2 (MRP2).

A crucial function of the liver is to produce bile, which aids in the digestion of lipids and in bilirubin excretion. Bile is secreted by distal hepatocytes into the canalicular space, followed by transport through a network of ducts of various sizes where it is further modified by cholangiocytes before being stored in the gallbladder for release into the duodenum (Figure 4A). Cholangiocytes, or biliary epithelial cells (BECs), are polarized cells that form a network of interconnected bile ducts with a high surface-to-volume ratio, enabling formation of an osmotic gradient and bile flow.

In cholangiocytes, intracellular calcium is released into the cytosol through InsP3R channels, of which the type III isoform (ITPR3) is most abundantly expressed (Figure 4B) (166). InsP3R channels are activated by multiple signaling pathways, including signaling via M3 muscarinic acetylcholine receptors (167), purinergic receptors P2 (P2X and P2Y) (168), as well as mechanical cues, such as bile flow and shear stress (169, 170). Flow induces calcium influx via channels in the cilium, including TRPV4 (171) and the polycystin-1–polycystin-2 complex (170). Elevations in cytosolic calcium levels activate the calcium-dependent chloride channel TMEM16A that mediates chloride-bicarbonate exchange at the apical side (172). TMEM16A can be further activated by flow, which is dependent on PKC-α and extracellular ATP binding to P2 receptors and increase in intracellular calcium (173).

Besides TMEM16A, CFTR constitutes the second important chloride efflux channel (174). CFTR signaling is triggered by binding of secretin to its receptor on the basolateral membrane, leading to stimulation of adenylate cyclase (AC) and formation of cAMP, which induces the translocation of vesicles containing AQP1, CFTR, and AE2 to the apical plasma membrane and subsequent phosphorylation of CFTR (175, 176). Notably, CFTR-expressing large duct cholangiocytes express the calcium-sensitive AC isoforms AC5, AC6, AC8, and AC9, whereas cAMP production in small ducts is mediated predominantly by the calcium-insensitive isoforms AC4 and AC7 (177). In addition to translocating chloride, CFTR has been suggested to act as an ATP release channel that reciprocally stimulates calcium signaling via the ATP-P2 receptor axis (174, 176). The role of CFTR in this apical ATP release remains controversial however, which may be due to an interaction between mechanical cues and CFTR that are as yet unresolved (178). Chloride efflux is partially dependent on parallel activation of apical chloride and basolateral potassium channel conductance, as well as potassium secretion, which is executed by IK-1 and SK2 channels (179, 180). Calcium activates those potassium channels, resulting in potassium efflux from cholangiocytes, enhanced chloride secretion, and hyperpolarization of the cell membrane.

While small intrahepatic bile ducts (IHBD) with a diameter < 15 μm express only the TMEM16A channels for chloride release, larger IHBD function through both TMEM16A and CFTR chloride release channels (172). The magnitude of ATP-stimulated chloride currents mediated by TMEM16A is 3x greater than stimulation by CFTR, which has led to the suggestion that TMEM16A is the predominant chloride efflux channel (181, 182).

Calcium signaling also plays important roles in bile secretion in hepatocytes where it modulates canalicular contractions. Increased calcium signaling promotes actin-myosin interactions (183) and enhances peristaltic contractions in pericentral to periportal direction (184). In hepatocytes, calcium is released upon stimulation with ATP, vasopressin, glucagon, and epinephrine (Figure 4C) (185, 186). Furthermore, calcium affects bile secretion by activation of the bile salt export pump (BSEP) transporter probably through enhancing its exocytic insertion, which is partially dependent on expression and pericanalicular localization of ITPR2 (187).

Cholestasis arises when bile flow is obstructed and is associated with both decreased calcium signaling in cholangiocytes and increased cytosolic calcium levels in hepatocytes (188). Expression of ITPR3 is reduced in many cholestatic diseases such as bile duct obstruction, biliary atresia, primary biliary cholangitis, and primary sclerosing cholangitis, resulting in diminished calcium signaling and calcium-mediated bicarbonate secretion (189). Importantly, downregulation of ITPR3 is specific to cholestatic conditions, as it is not seen in hepatitis C viral infection, which is associated with inflammation but not cholestasis *per se* (189). Although the mechanisms leading to ITPR3 downregulation have not been determined, several explanations have been proposed, including FXR-mediated repression of *ITPR3* by bile acids, proinflammatory cytokine repression of fluid secretion and ITPR3, as well as reduced synthesis or increased degradation of ITPR3 (188). Similar effects on calcium signaling have been reported for loss of pericanalicular ITPR2, which is downregulated in estrogen and endotoxin models of cholestasis (187). In hepatocytes, an increase in cytosolic calcium levels due to vasopressin or by the bile acids taurolithocholate and lithocholic acid inhibits bile secretion and canalicular peristaltic waves necessary for maintaining bile flow, giving rise to cholestasis (190). It has been suggested that cytosolic calcium inhibits actin filaments from contracting to induce peristaltic waves, thus leading to cholestasis.

Mutations in the *ABCB11* gene, which encodes the BSEP transporter, can cause progressive familial intrahepatic cholestasis type 2 (PFIC2). *ABCB11* mutations lead to decreased bile salt secretion followed by bile salt accumulation and hepatocyte damage (191). While the role of calcium signaling in PFIC2 progression has not been directly demonstrated, calcium is required for BSEP activity (187) and calcium depletion rapidly leads to cholestasis in liver explants (192). Further, growing evidence suggests that genetic alterations in *ABCB11* may predispose individuals to drug-induced cholestasis (193). It is therefore essential to examine calcium signaling at both the single cell and overall tissue level to fully understand the role of calcium signaling in cholestasis. Emerging single cell data sets may be able to provide more detail on how stimuli translate into nuclear, cytosolic, or extracellular calcium signals, and why cholangiocytes and hepatocytes tend to display different calcium profiles in response to similar stimuli.

Cholestatic conditions can be treated with ursodeoxycholc acid (UDCA) and its taurine conjugate derivate TUDCA, of which the former is approved by the FDA for the treatment of primary biliary cirrhosis. Both ambiphilic bile acids stimulate BSEP integration in canalicular membranes, increasing exocytosis, ATP release, intracellular calcium levels, membrane chloride permeability, and transepithelial secretion in cholangiocytes via both chloride channels TMEM16A and CFTR (174, 181, 194, 195). UDCA also induces calcium release and secretion of ATP into bile by hepatocytes, which in turn activates calcium signaling in cholangiocytes via P2 receptors, thus facilitating bile flow (196, 197). In summary, cholestasis is characterized by impaired calcium signaling and restoration of calcium homeostasis by UDCA treatment is paralleled by an amelioration of symptoms and restoration of bile flow. For more details on diagnosis and treatment of cholestatic liver diseases we refer the interested reader to the clinical practice guidelines of the European Association for the Study of the Liver (198, 199).

## Conclusions

Calcium is a versatile second messenger that plays essential roles in a plethora of hepatic processes. Direct perturbations of mitochondrial calcium levels are common features of acute liver injury, as in DILI and IRI, leading to increased ROS formation, mitochondrial depolarization and, eventually, liver cell apoptosis. In contrast, alterations of cytosolic calcium signaling accompanied by the depletion of ER calcium, ER stress as well as activation of UPR and is a common hallmark of multiple chronic liver diseases, including NAFLD and cholestasis. Moreover, ER stress directly causes insulin resistance, resulting in progressive metabolic dysregulation. Initially, mitochondria can efficiently buffer acute perturbations of cytosolic calcium levels; however, this capacity becomes overloaded under chronic conditions. Thus, mitochondrial injury due to calcium perturbations constitutes a secondary effect in chronic liver diseases.

Importantly, liver cells specifically regulate calcium signaling in different cellular compartments. Nuclear calcium transients are elicited by translocation of various receptor tyrosine kinases from the cytoplasmic membrane into the nucleus, followed by activation of nuclear PLCγ and opening of the InsP_3_R calcium channels within the nucleoplasmic reticulum. So far, nuclear calcium signaling in the liver has been exclusively reported in the context of liver regeneration, specifically signaling through the HGFR, EGFR, and InsR.

Modulation of calcium signaling constitutes an emerging strategy in the treatment of various acute and chronic liver insults. While the therapeutic focus in IRI and DILI is on the prevention of MPTP opening, explored treatment opportunities for chronic liver disease are mechanistically more diverse. Treatment of cholestasis generally aims at a stimulation of intracellular calcium signaling to stimulate bile flow. In contrast, a lowering of cytosolic calcium levels is intended in the treatment of metabolic liver disease. To this end, therapeutic targets include the redistribution of cytoplasmic calcium into the ER by stimulation of SERCA, as well as the amelioration of ER stress by chemical chaperones. While these approaches have yet to make their way into primary care, auspicious results from clinical trials suggest that modulation of calcium signaling constitutes a promising step forward in the treatment of chronic liver diseases.

## Author contributions

All authors listed have made a substantial, direct and intellectual contribution to the work, and approved it for publication.

### Conflict of interest statement

VL is a co-founder and owner of HepaPredict AB. The remaining authors declare that the research was conducted in the absence of any commercial or financial relationships that could be construed as a potential conflict of interest.
